# Early results of a safety and feasibility clinical trial of a novel single-port flexible robot for transoral robotic surgery

**DOI:** 10.1007/s00405-017-4729-y

**Published:** 2017-09-04

**Authors:** Jason Y. K. Chan, Eddy W. Y. Wong, Raymond K. Tsang, F. Christopher Holsinger, Michael C. F. Tong, Philip W. Y. Chiu, Simon S. M. Ng

**Affiliations:** 1 Department of Otorhinolaryngology, Head and Neck Surgery, Prince of Wales Hospital, The Chinese University of Hong Kong, Shatin, N.T., Hong Kong SAR, China; 2 Department of Surgery, The University of Hong Kong, Pok Fu Lam, Hong Kong SAR, China; 3 Department of Otorhinolaryngology, Head and Neck Surgery, Stanford University, Palo Alto, USA; 4 Department of Surgery, The Chinese University of Hong Kong, Shatin, N.T., Hong Kong SAR, China

**Keywords:** Flexible robot, Transoral robotic surgery, TORS, da Vinci, Clinical trial, Transoral

## Abstract

The aim of this study was to describe the early results of a phase 1 safety and feasibility clinical trial of the first clinical use of a novel robot for transoral robotic surgery (TORS)—the da Vinci SP (Intuitive Surgical Inc., Sunnyvale, CA, USA). Study design of this study is prospective clinical trial. The methods used in this study are prospective innovation, development, exploration, assessment, and long-term study phase 1 clinical trial. Early results of six patients underwent TORS with the da Vinci SP (Intuitive Surgical Inc., Sunnyvale, CA, USA) demonstrate access the nasopharynx, oropharynx, larynx, and hypopharynx. There were no conversions of the robotic surgical system. There were no serious adverse events or adverse events related to the use of the robot at 30-day follow-up for all six patients. The early results of this safety and feasibility trial of the da Vinci SP (Intuitive Surgical Inc., Sunnyvale, CA, USA) clearly demonstrate that the device is safe and that it is feasible in performing TORS to access the nasopharynx, oropharynx, larynx, and hypopharynx.

**Electronic supplementary material:**

The online version of this article (doi:10.1007/s00405-017-4729-y) contains supplementary material, which is available to authorized users.

## Introduction

Since the first description of a novel, flexible, single-arm robotic surgical system (da Vinci SP Surgical system, Model SP999; Intuitive Surgical Inc.) was first evaluated clinically in 2010 on 19 patients undergoing urological surgery [1]. Further technical refinements have been developed in this robotic system with recent publications containing a detailed description of the current design of the robot and cadaveric applications in transoral robotic surgery (TORS) [2, 3]. In brief, this next generation system incorporates a stereoscopic binocular camera and three 6 mm flexible instruments in a cannula of 2.5 cm diameter. The flexible robotic arms allow the instruments to be deployed from the oral cavity to the nasopharynx, oropharynx, hypopharynx, and larynx. Here, we describe the early results of the first ever clinical investigation into the feasibility and safety of this updated flexible single-arm robot, da Vinci SP (Intuitive Surgical Inc.), in TORS.

## Methods

### Study design

This was a prospective institutional review board approved, innovation, development, exploration, assessment, long-term study (IDEAL) phase 1 clinical trial conducted in accordance with the Declaration of Helsinki under the institutional review board and local regulatory body approval of The Chinese University of Hong Kong. The study was registered on http://www.ClinicalTrials.gov (NCT03010813).

### Study population

Both benign and malignant lesions of the head and neck were included with the first six cases, as shown in Table 1.

**Table 1:** Demographics, diagnosis, and procedure of the first six cases for transoral robotic surgery utilizing the da Vinci SP

Case	Gender		Age	Previous radiotherapy	Diagnosis	Procedure	Retractor	Time to exposure (min)	Time to dock robot (min)	Estimated blood loss (ml)
1	M	72		Yes	Tongue base papilloma	TORS excision of the tongue base	CD	16	3	5
2	F	52		Yes	Second primary T1 oropharyngeal SCC	TORS examination under anaesthesia of the OP and NP	Dingman	10	4	5
3	M	53		Yes	Recurrent T2 Supraglottic Carcinoma	TORS examination under anaesthesia of the larynx	CD	43	1	0
4	M	75		Yes	Pharyngeal stricture	TORS dilatation of pharyngeal stricture	CD	4	3	10
5	M	64		No	Supraglottic inflammatory mass	TORS excision of supraglottis	FK	35	5	50
6	M	54		No	Unknown primary SCC, P16 +ve	TORS excision of the tongue base	CD	20	4	20

*M* male, *F* female, *SCC* squamous cell carcinoma, *OP* oropharynx, *NP* nasopharynx, *CD* Crowe–Davis, *FK* Feyh–Kastenbauer

### Study endpoints

Primary endpoint included conversion rates and perioperative complications within 30 days following surgery. Secondary endpoints included operative time, blood loss, and pain scores as documented with a visual analogue scale.

### Statistical analysis

The Fisher’s exact test and Mann–Whitney *U* test will be used to compare categorical, and non-parametric data for the trial. A *p* value <0.05 was considered to be statistically significant.

## Results

**Fig. 1:**
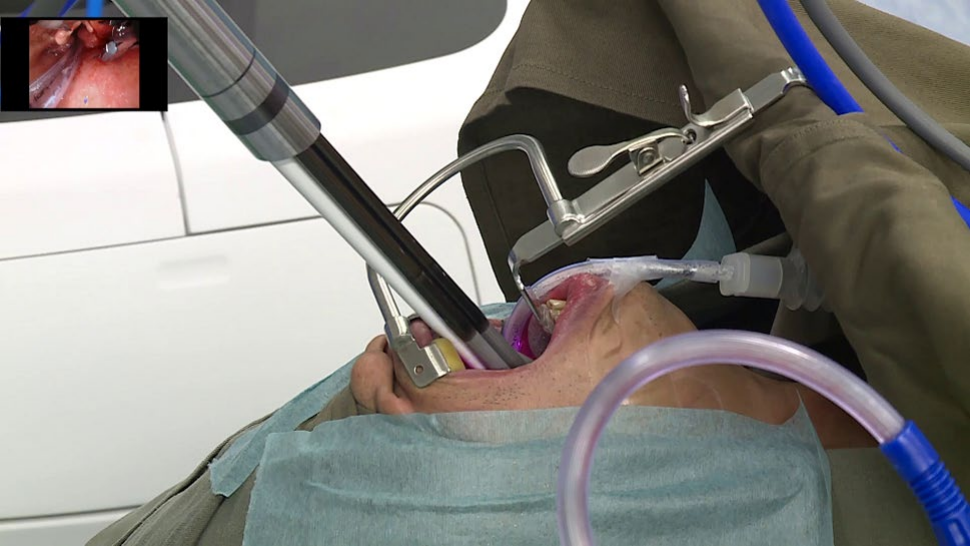
da Vinci SP in use for examination of the supraglottis with the camera arm and all three instrument arms deployed transorally as seen in the insetted figure. The mouth is opened and suspended with a Crowe–Davis mouthgag. The cannula is located about 10 cm from the oral opening

**Fig. 2:**
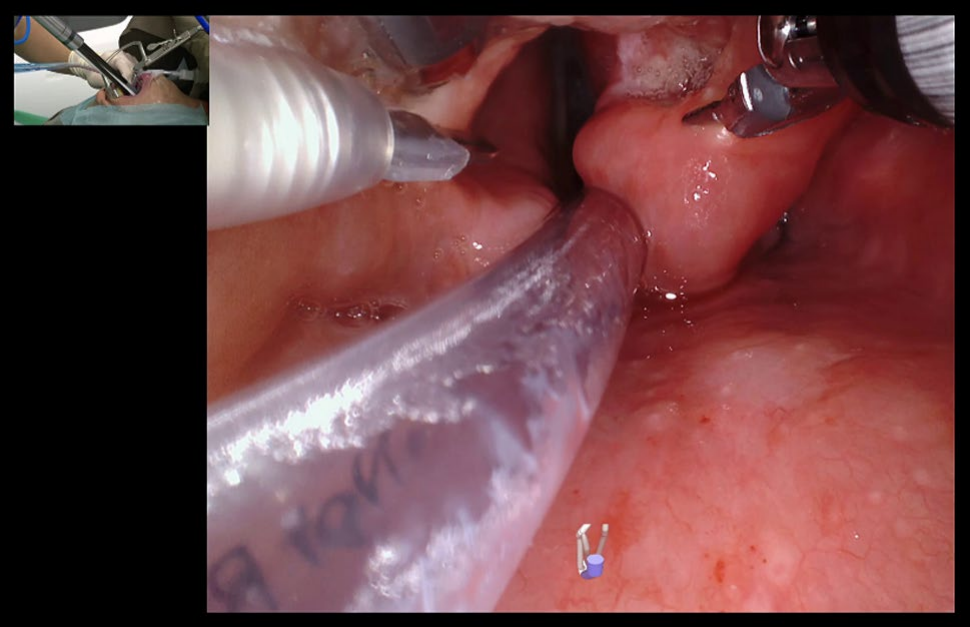
da Vinci SP in use for examination of the supraglottis through an internal view. The monopolar spatula tip is seen on the left, the Maryland bipolars are opened at the right aryepiglottic fold exposing the tumor, while the fenestrated bipolar is retracting the epiglottis superiorly. The insetted figure shows the assistant holding a Yankaur suction and the location of the port about 10 cm from the oral opening

Among the first six enrolled patients, there were no conversions to alternative surgical approaches. Figures 1, 2 show the da Vinci SP (Intuitive Surgical Inc., Sunnyvale, CA) being utilized in case 3 to examine the larynx (Supplementary video 1). The procedures underwent are listed in Table 1, demonstrating that with the da Vinci SP (Intuitive Surgical Inc., Sunnyvale, CA, USA) we could reach the nasopharynx, oropharynx, hypopharynx, and larynx utilizing even just a simple daily Crowe–Davis retractor. The main use of time was in obtaining adequate exposure with the initial use of the da Vinci SP (Intuitive Surgical Inc., Sunnyvale, CA, USA), mean 21.3 min (range 4–43 min). The initial time to dock the robot was negligible with a mean of 3.3 min (range 1–5 min). There were no conversions of the robotic surgical system. Mean visual analogue scale pain scores preoperatively, at 2 week and 30 day postop, were 0.16 (range 0–1), 0.67 (range 0–4), and 0.16 (range 0–1). There were no serious adverse events or adverse events related to the use of the da Vinci SP (Intuitive Surgical Inc., Sunnyvale, CA, USA). There were surgical complications in two patients unrelated to the robot use. Case number four, a patient who had previous radiotherapy and poor dentition had a fractured right lower molar that required a dental consultation. Case number five had a 1 cm laceration of the tongue from the mouthgag that resolved by itself on follow-up visits.

## Discussion

The early results of this safety and feasibility trial of the da Vinci SP (Intuitive Surgical Inc., Sunnyvale, CA, USA) clearly demonstrate that the device is safe and that it is feasible in performing transoral surgery to access the nasopharynx, oropharynx, larynx, and hypopharyngeal region. This has been achieved in a predominantly Southern Chinese population despite significantly different, in particular smaller cranio-facial measurements as compared to Caucasians [4].

A significant advantage offered by the da Vinci SP (Intuitive Surgical Inc., Sunnyvale, CA, USA) is the option to use three instruments to perform transoral endoscopic surgery. The working space within the instruments and camera is roughly the size of a tennis ball. This third arm replaces the need of an assistant to provide traction and counter traction and allows the surgeon to control these maneuvers to better dissect critical neurovascular structures. Other distinct advantages include the flexible camera enabled with digital zoom that allows magnification and easier visualization of lesions and the different compartments of the pharynx.

There are some limitations to the system; now, with the extra arm, there is less working space for an assistant to, in particular, aid with suction. However, this can be largely overcome with a suction catheter placed through the nasal cavity into the desired location. Another limitation is the lack of bone instrumentation to resect, in particular tumors of the nasopharynx and skull base.

## Conclusion

The early results of the use of the novel single-port flexible robotic system, the da Vinci SP (Intuitive Surgical Inc., Sunnyvale CA, USA) demonstrate that the device is safe and feasible to use for TORS to access the nasopharynx, oropharynx, larynx, and hypopharynx.

## Electronic supplementary material

Below is the link to the electronic supplementary material.

Supplementary material 1 (AVI 74010 kb)
